# Squamous cell carcinoma with sarcomatous transformation of the penis

**DOI:** 10.4322/acr.2021.303

**Published:** 2021-08-20

**Authors:** Joana dos Santos, Rafael Cabrebra, Beatriz Neves, Eduardo Silva, António Polónia

**Affiliations:** 1 Unidade Local de Saúde de Matosinhos (ULSM), Department of Pathology, Matosinhos, Porto, Portugal; 2 Instituto Português de Oncologia de Lisboa (IPO Lisboa), Department of Pathology, Lisboa, Lisboa, Portugal; 3 Universidade do Porto (U. Porto), Institute of Molecular Pathology and Immunology (IPATIMUP), Ipatimup Diagnostics, Porto, Porto, Portugal; 4 Instituto de Investigação e Inovação em Saúde (I3S), University of Porto, Porto, Porto, Portugal; 5 Instituto Português de Oncologia de Lisboa (IPO Lisboa), Department of Urology, Lisboa, Lisboa, Portugal

**Keywords:** squamous cell carcinoma, verrucous carcinoma, sarcomatoid carcinoma, penile neoplasms

## Abstract

Malignant tumors of the penis are rare, most of them being squamous cell carcinomas (SCCs). We report the case of a 75-year-old man with a large penile mass submitted to partial penectomy. The specimen showed an exophytic mass involving the glans, coronal sulcus, and prepuce. Microscopic examination showed a carcinoma with two distinct areas: a mixed SCC and a sarcomatoid carcinoma. The SCC component had areas of verrucous carcinoma and areas of classical invasive SCC. The tumor cells expressed p63 with the absence of p16 expression. Vimentin and p53 were positive in the sarcomatous component. The morphology and immunohistochemistry were compatible with mixed SCC (verrucous hybrid-sarcomatoid carcinoma). Additionally, the tumor cells also expressed 3 different clones of PDL1 (22C3, SP263, and SP142). Two months later, the patient presented local recurrence with multiple lymph nodes and lung metastases, dying 7 weeks later. Mixed tumors represent diagnostic challenges. The correct identification of adverse prognostic factors can be the first step to implement the treatment with a higher probability of success.

## INTRODUCTION

Malignant tumors of the penis are rare, although incidence rates are variable amongst different countries, probably related to environmental factors such as socioeconomic deprivation, poor hygiene, phimosis, lichen sclerosus, and human papillomavirus (HPV) infection.^1^^,^ 2 ^,^ 3

Most penile cancers are squamous cell carcinomas (SCCs) arising in the glans, foreskin, and coronal sulcus, in this order of frequency. It usually presents as an exophytic mass or a non-ulcerated lesion, mainly affecting patients in their fifth and sixth decade of life.^1^^,^ 2There are several subtypes of penile SCC which are important to recognize since they have different clinical, morphological, and prognostic features. 2 ^,^ 4 The verrucous, papillary, and warty, usual and mixed are low-risk morphological variants. On the contrary, sarcomatoid, basaloid, and adenosquamous are considered high-risk. 2 ^,^ 4

These subtypes are also divided by two pathogenic pathways: one is related to high-risk HPV, and the other has non-HPV-related pathogenesis.^1^^,^ 2

For that reason, WHO subclassification of SCCs is based on the relation with HPV infection and clinicopathological features. Furthermore, the non-HPV-related tumors can be divided into two groups: one with TP53 mutation (usually aggressive) and another with high chromosomal instability.^2^

A minority of these tumors can show a mixed pattern. The most frequent association is of warty and basaloid tumors. 2 ^,^ 4

Penile cancer demands aggressive treatment with partial/total penectomy.^6^ The most important prognostic factors are the clinical stage, histological subtype and grade, pattern of invasion, peri-neural/vascular invasion and lymph node metastasis. Most recurrences develop in the first five years after surgery. 7 ^,^ 8

## CASE REPORT

**Figure 1:**
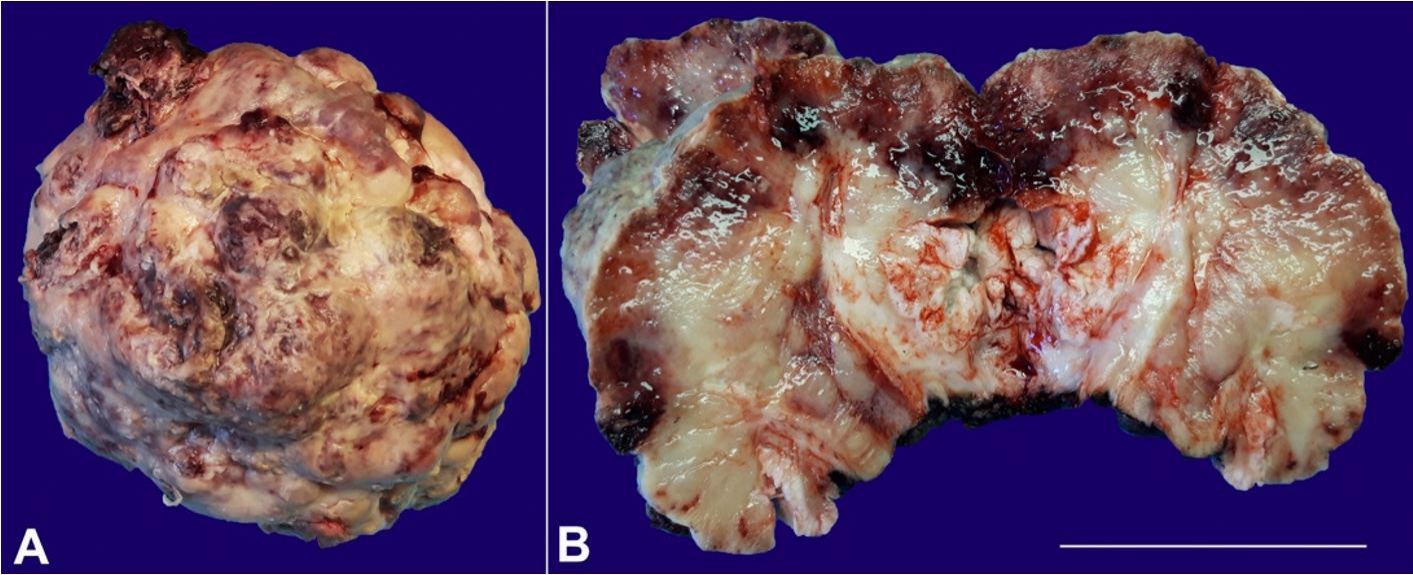
**A** – Macroscopic feature of the partial penectomy showing a large exophytic mass with an irregular surface; **B** – Macroscopic cross-section of the partial penectomy showing a gray, white and congestive tissue. Scale bar = 70mm.

A 75-year-old man underwent partial penectomy as a consequence of a large penile mass. Grossly, an exophytic and gray mass with 9.0x8.2x7.5cm was identified, involving the glans. The cut surface was gray and white with congestive areas (Figure 1).

**Figure 2:**
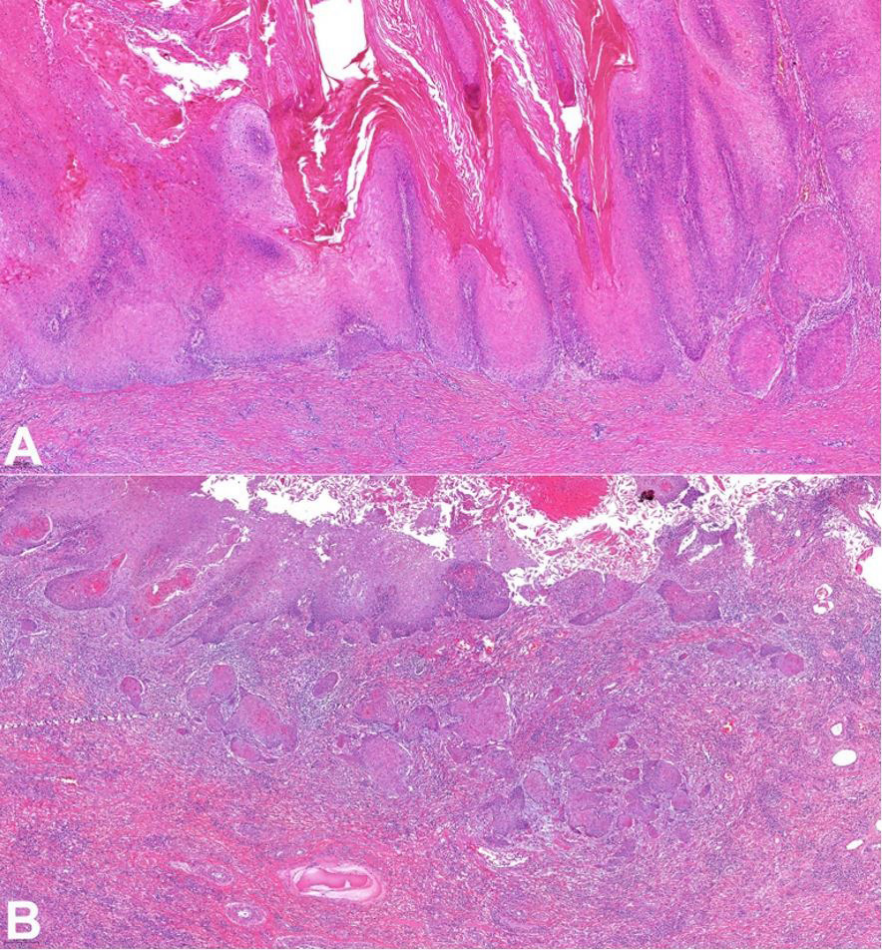
Photomicrographs of the tumor. **A** – Pure verrucous carcinoma component (HE 100x); **B** - Focus of usual invasive squamous cell carcinoma (verrucous hybrid carcinoma) (HE 100x)

On the microscopic examination, the lesion showed two distinct areas. One area showed papillomatosis, with papillae of variable length without prominent fibrovascular cores. This component was well-differentiated, with cells showing bland, small, and round nuclei. The cells did not show koilocytosis. There was extensive hyper-parakeratosis and acanthosis. The tumor front was regular, broad, and pushing with a small focus of invasion (usual SCC type), consistent with verrucous hybrid carcinoma (Figure 2).

**Figure 3:**
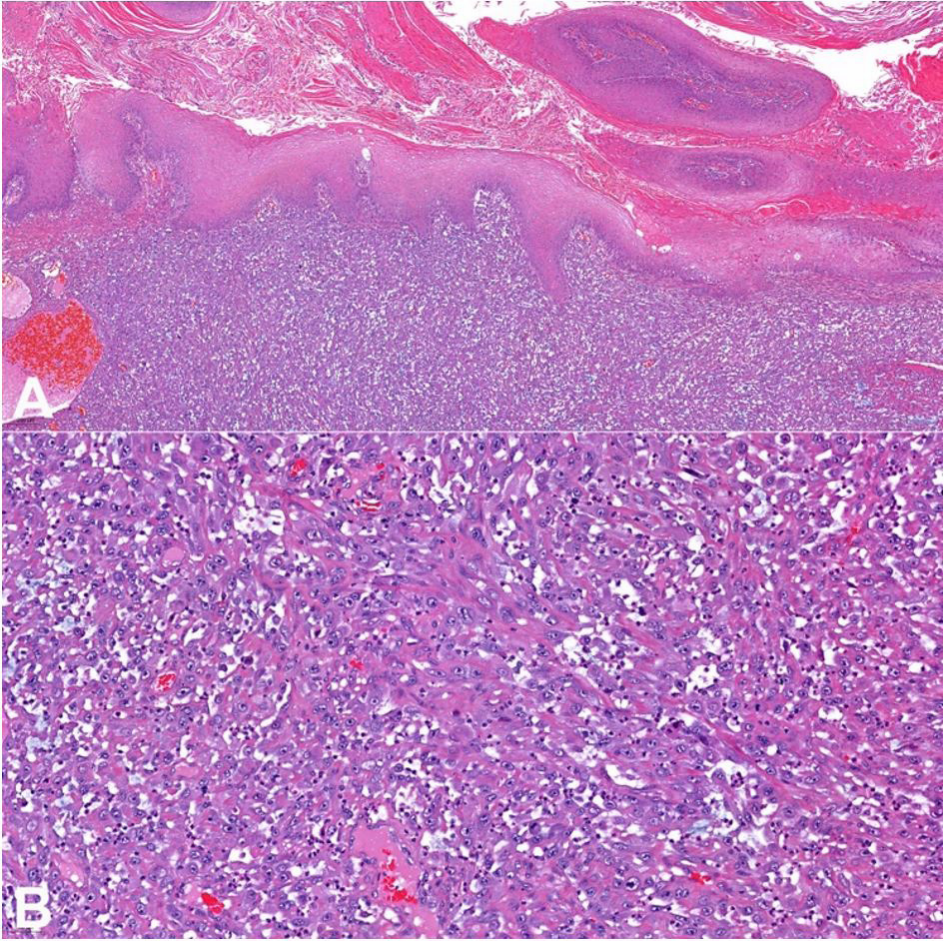
Photomicrographs of the tumor. **A** – Sarcomatous component adjacent to verrucous carcinoma (HE 100x); **B** – Sarcomatous component (HE 200x).

The other component was composed of atypical spindle cells arranged in vascularized bundles, mostly discohesive (Figure 3). These cells had enlarged and pleomorphic nuclei with conspicuous nucleoli and amphophilic cytoplasm. Mitoses were frequent, sometimes atypical, as well as areas of necrosis. The lymphovascular invasion was observed without perineural invasion. An inflammatory infiltrate with polymorphonuclear leukocytes and lymphocytes was also observed within the tumor. There was no mesenchymal differentiation. The morphologic features were consistent with a penile mixed SCC (verrucous hybrid-sarcomatoid carcinoma). The tumor compromised the cavernosum and spongiosum corpus. Surgical margins were free from tumor.

**Figure 4:**
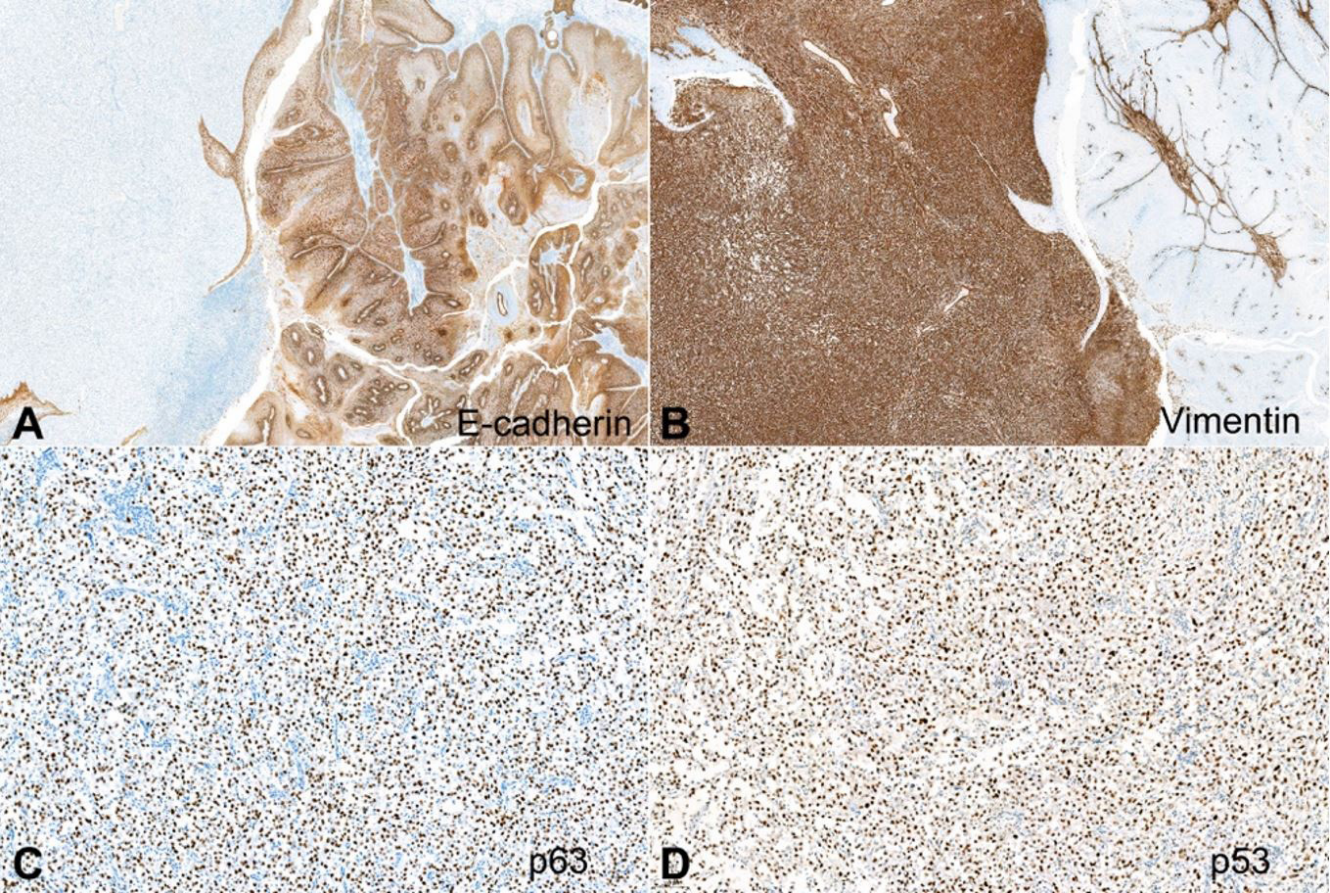
Photomicrographs of the tumor. **A** – Positive E-cadherin expression in the verrucous carcinoma component (right) and negative in the sarcomatous component (left) (100x); **B** – Positive vimentin expression in the sarcomatous component (100x); **C** – Positive p63 expression in the sarcomatous component (100x); **D** – Positive p53 expression in the sarcomatous component (100x).

Immunohistochemistry staining showed a strong expression of p63 with the absence of p16 expression in both components. Vimentin and p53 were positive only in the sarcomatous component as well as loss of E-cadherin expression (Figure 4).

**Figure 5:**
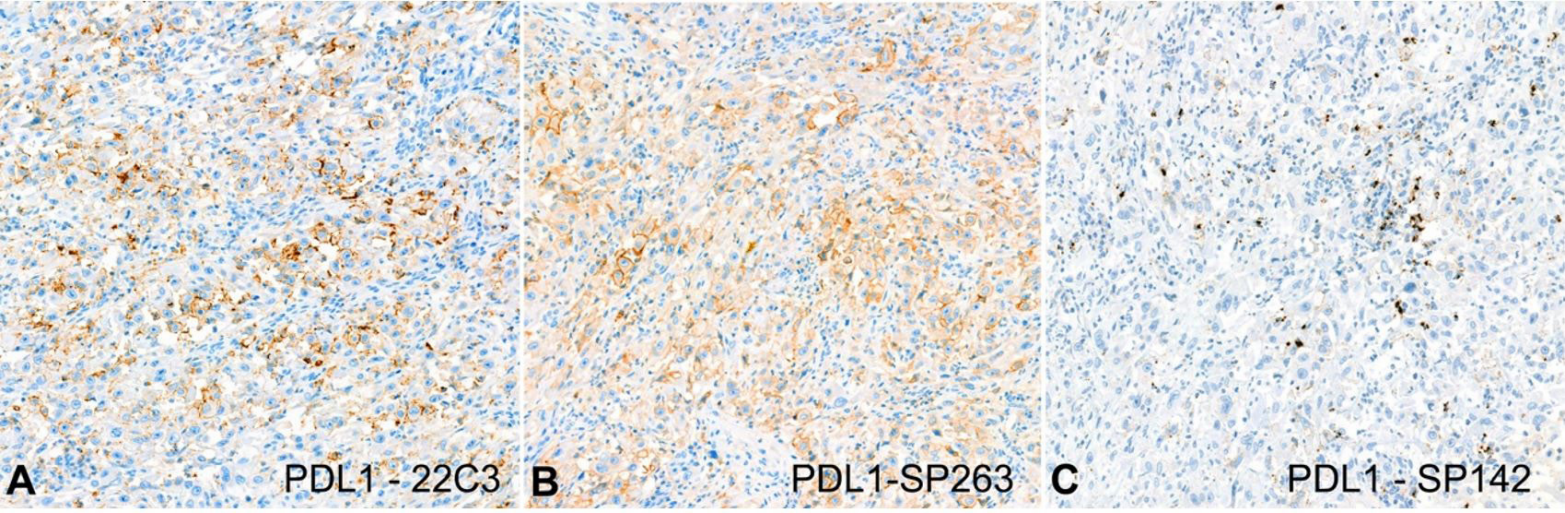
Photomicrographs of the tumor. PDL1 expression **A** – Positive expression for 22C3 (200x); **B** – Positive expression for SP263 (200x); **C** – Positive expression for SP142 (200x).

Additionally, we evaluated the expression of PD-L1 with 3 different clones of antibodies (22C3, SP263, and SP142). The combined positive score (CPS) was 30% and 15% for the 22C3 and SP263 clones, respectively, and the inflammatory score (IC) was 5% for the SP142 clone (Figure 5). There was no pan-TRK staining in either component.

Two months later, the patient was admitted with sepsis and a voluminous lesion on the remaining penis. Imaging studies showed multiple nodules in the lungs as well as lymphadenopathies in the inguinal, mediastinal, and iliac regions. Total penectomy with perineal urethrostomy was performed one month later. The gross examination of the remaining penectomy revealed a mass with extensive necrosis and involvement of corpus cavernosum. Histologically, a sarcomatoid squamous cell carcinoma was observed with vascular and perineural invasion, and involvement of surgical margins. The patient died 21 days after the total penectomy.

## DISCUSSION

Mixed carcinomas are defined by the presence of two or more variants of SCC in the same tumor, generally affecting older patients in the seventh decade of life.^2^

Verrucous carcinoma is a rare tumor. HPV is usually absent, and koilocytosis is not present.^1^Microscopically, verrucous carcinoma is well-differentiated, showing minimal atypia, papillomatosis, hyperkeratosis, acanthosis, and a broad-based interface between the tumor and stroma.^1^There may be a dense lymphocytic infiltrate in the stroma. Squamous hyperplasia, differentiated penile intraepithelial neoplasia, and lichen sclerosus are frequently found at the lesion‘s periphery.^1^^-^ 5 Verrucous carcinomas may be associated with other variants, most frequently with usual SCC, as well as the sarcomatoid variant, especially after radiation therapy.^1^ In this case, there was no previous exposure to radiation therapy. Verrucous carcinomas require thorough sectioning to exclude foci of higher-grade SCC since these components will drive the patient’s prognosis.^5^

On the other hand, sarcomatoid SCC is an aggressive, rare, and non-HPV-related neoplasm composed predominantly of spindled cells, sometimes with heterologous focal elements (muscle, bone, or cartilage). Regional metastases are very frequent, and this tumor is associated with high mortality.^1^^,^ 5

Regarding immunotherapy, recent studies have found the expression of PD-L1 in 40% of the penile SCC. This finding shows a potential therapeutic advantage since there is evidence that the use of anti-PD-1/PD-L1 agents may be beneficial in metastatic penile SCC treatment. However, further investigation is needed to clarify this therapeutic potential.^5^^,^ 9 ^,^ 10 ^,^ 11

First-generation TRK inhibitors show high response rates in NTRK fusion-positive cancers regardless of tumor histology, although there are no studies regarding penile SCC specifically.^12^

The epithelial–mesenchymal transition (EMT) is associated with aggressive penile SCC subtypes, but not with the presence of HPV. During this process, the epithelial cells lose membranous E-cadherin and gain vimentin expression.^13^^,^ 14 Therefore, vimentin and E-cadherin could be used as prognostic markers. HPV infection is also associated with loss of membranous E-cadherin.^13^^-^ 16

Our case presented two opposing elements, an extremely well-differentiated carcinoma, and a sarcomatous component. Despite the adequate surgical margins obtained in the initial partial penectomy specimen, the vascular invasions as well as cavernosum and spongiosum corpora invasion, loss of E-cadherin expression, vimentin, and p53 positivity in the sarcomatous component was indicative of an aggressive tumor, which is associated with high-risk of nodal and distant metastasis as well as high-rate mortality.^4^^,^ 5 ^,^ 17 The sarcomatous component was responsible for the clinical behavior of the disease, as shown by the exclusive presence of this component in the recurrence.

## CONCLUSION

Mixed tumors represent a diagnostic challenge since it may be difficult to identify the coexistence of more than one histological pattern. Importantly, proper tumor sampling and strict morphological criteria should aid in the histologic evaluation. The identification of adverse prognostic factors should be the basis for an aggressive initial therapy to prevent recurrence.^5^^,^ 6
